# Detecting Burn Severity and Vegetation Recovery After Fire Using dNBR and dNDVI Indices: Insight from the Bosco Difesa Grande, Gravina in Southern Italy

**DOI:** 10.3390/s25103097

**Published:** 2025-05-14

**Authors:** Somayeh Zahabnazouri, Patrick Belmont, Scott David, Peter E. Wigand, Mario Elia, Domenico Capolongo

**Affiliations:** 1 Department of Earth and Geo-Environmental Sciences, University of Bari Aldo Moro, 70121 Bari, Italy; 2 Department of Watershed Sciences, Utah State University, Logan, UT 84322, USA; patrick.belmont@usu.edu (P.B.); scott.david@usu.edu (S.D.); 3 Division of Earth and Ecosystem Sciences, Desert Research Institute, Reno, NV 89512, USA; pewigand@gmail.com; 4 Department of Agricultural and Environmental Sciences, University of Bari Aldo Moro, 70121 Bari, Italy; mario.elia@uniba.it

**Keywords:** wildfire, burn severity, vegetation recovery, Google Earth engine, vegetation indices, Bosco Difesa Grande

## Abstract

Wildfires serve a paradoxical role in landscapes—supporting biodiversity and nutrient cycling while also threatening ecosystems and economies, especially as climate change intensifies their frequency and severity. This study investigates the impact of wildfires and vegetation recovery in the Bosco Difesa Grande forest in southern Italy, focusing on the 2017 and 2021 fire events. Using Google Earth Engine (GEE) accessed in January 2025, we applied remote sensing techniques to assess burn severity and post-fire regrowth. Sentinel-2 imagery was used to compute the Normalized Burn Ratio (NBR) and Normalized Difference Vegetation Index (NDVI); burn severity was derived from differenced NBR (dNBR), and vegetation recovery was monitored via differenced NDVI (dNDVI) and multi-year NDVI time series. We uniquely compare recovery across four zones with different fire histories—unburned, single-burn (2017 or 2021), and repeated-burn (2017 and 2021)—providing a novel perspective on post-fire dynamics in Mediterranean ecosystems. Results show that low-severity zones recovered more quickly than high-severity areas. Repeated-burn zones experienced the slowest and least complete recovery, while unburned areas remained stable. These findings suggest that repeated fires may shift vegetation from forest to shrubland. This study highlights the importance of remote sensing for post-fire assessment and supports adaptive land management to enhance long-term ecological resilience.

## 1. Introduction

Wildfires serve an important function in many ecosystems, helping to maintain ecological balance, promote biodiversity, and aid in natural regeneration. In ecosystems predisposed to recurrent fire, such as certain woods and grasslands, flames sweep out dead matter, reduce competition, and recycle nutrients, providing circumstances that stimulate new growth [1,2]. Although wildfires are an important part of the Earth’s ecosystem, they can also result in significant economic losses, severe air pollution, human fatalities, and environmental harm. Human activities and climate change are having an increasing impact on fire regimes [3,4]. Anthropogenic climate change has significantly increased the frequency, intensity, and size of wildfires. Warmer temperatures, drier conditions, and increased wind lead to more extreme fire weather, facilitating faster and more intense fire spread [5]. Recent research has shown the global frequency and magnitude of intense wildfires have more than doubled since 2003. Additionally, extreme fires have increased significantly in regions such as temperate conifer forests, boreal forests and in Calabria, Italy [6]. Six of the seven severe wildfire years have occurred since 2017 [7,8].

In addition to general climatic trends, specific climate phenomena like El Niño have also been shown to influence wildfire dynamics. A recent study [9] used the SPEEDY-HYCOM model to analyze the impact of different El Niño intensities on wildfire risk across Africa. By applying the Meteorological Fire Danger Index (MFDI), the study demonstrated that even weak and moderate El Niño events significantly affect fire hazards with varying temporal lags, while strong events showed high fire risk consistently across East Africa [10]. These findings highlight the need to consider both global warming and interannual climate variability, such as El Niño, in wildfire risk assessments and management strategies [11]. Fire patterns have historically been influenced by climate variability, with fires being more common in drier, warmer climates [12]. Fire pattern refers to the spatial and temporal distribution of wildfires, including their frequency, size, intensity, and spread dynamics, which are affected by elements such as climate, vegetation, and topography [13]. Recent research based on sedimentary charcoal data in central Italy demonstrates the presence of high-frequency fire periods, lasting 300–500 years, related to dry summers through the Holocene [14]. The Mediterranean region has historically experienced significant wildfires, accounting for 90% of Europe’s total burned area [15]. The Mediterranean climate’s extended drought and high summer temperatures are distinguishing factors that define the temporal and spatial limitations of the major fire season [16]. According to research [17], under global warming scenarios of 1.5 °C, 2 °C, and 3 °C, the summer burned area in Mediterranean Europe could increase by 40% to 100%; yet, limiting global warming to well below 2 °C, as per the Paris Agreement, would significantly reduce fire-related risks.

Fire is essential for vegetation regeneration because it removes dead material, recycles nutrients, and creates circumstances conducive to new plant growth. However, human activities such as fire suppression and land-use changes have altered natural fire cycles, resulting in thicker forests with higher fuel loads and an increased risk of catastrophic wildfires [18]. The negative impression of fire might overshadow the importance of better forest management methods, such as planned burns and sustainable land use, which contribute to ecological balance [19]. Many plant species in fire-prone habitats adapt to specific fire regimes that correlate to historical conditions, and movements beyond these limits can have negative impacts on vegetative regrowth and long-term species persistence [20]. The rate of vegetation regeneration following a wildfire varies significantly based on fire severity, ecosystem type, and other factors such as climate and soil conditions [21]. Landscapes and vegetation are naturally variable over time, constantly adjusting to variations in climate and other factors [22]. High-severity wildfires cause extreme alterations in vegetation cover and land surface properties [23,24]. In a study examining post-fire regeneration in the western United States, researchers analyzed vegetation recovery following the 2000 Storrie Fire, which burned approximately 23,000 hectares in northern California. Using a stratified sampling design, they assessed the impacts of burn severity and forest type on tree seedling regeneration, shrub cover, and overstory dynamics almost ten years after the fire. Their findings underscore the role of fire severity and site-specific abiotic conditions in shaping long-term ecological recovery processes [25]. Similarly, a recent study in the western Mediterranean evaluated post-fire vegetation regeneration across 200,000 hectares affected by 268 wildfires between 1988 and 2015. Leveraging Landsat imagery and the Tasseled Cap Transformation Brightness (TCTB) index, researchers tracked recovery and applied Random Forest models to analyze the influence of climate, fire severity, and terrain across an aridity gradient. The study identified drought duration as the primary factor limiting vegetation recovery; its suppressive effect was strongest in both semi-arid and humid zones. By comparison, the influence of burn severity on regrowth varied across the aridity gradient, with severity effects differing between dry and wetter sites. In contrast, topography showed minimal influence on post-fire regeneration [26]. More severe fires cause greater vegetation damage and carbon emissions, they can also accelerate post-fire plant regrowth and carbon sequestration, demonstrating intricate relationships between burn severity and ecological processes [27]. Many plant species in fire-prone locations have evolved to withstand specific fire regimes, and deviations from these regimes can have a severe influence on their long-term survival and regeneration.

Monitoring vegetation conditions and functioning with remote sensing is essential to understanding terrestrial vegetation status, phenology, and health on a global scale by deriving data that is both spatially explicit and resolved over time [28]. Although remotely sensed derived spectrum metrics offer no information on post-fire vegetation structural changes, they are a useful tool for tracking spectral vegetation changes and contributing to time-series analyses of post-fire vegetation recovery [29].

Vegetation indices, particularly NDVI, serve as indicators of vegetation growth and ecosystem change [30,31]. NDVI is widely used for various practical applications, including burn and vegetation regrowth monitoring [32], and it ranks as the most utilized index for assessing vegetation health and density [31]. There is evidence in some places that NDVI can capture vegetation post-fire trends [31]; however, in other locales, such as Mediterranean or boreal forests, additional satellite analysis and field data are needed to test its accuracy and applicability deeper [33]. The NDVI is sensitive to the first signs of canopy greening and the early stages of vegetation recovery; however, it saturates when a dense grass cover dominated by herbaceous pioneer species establishes on newly burned soil, causing it to be less effective as an alternative for post-fire vegetation structure and tree cover in some regions [34]. The post-fire vegetation recovery duration can vary depending on a variety of factors such as vegetation type, fire severity, post-fire weather events, and ecoregion. Recovery times range from a couple of years for Mediterranean shrublands with resprouting potential to decades in boreal forests [35]. Researchers commonly use NBR as a vegetation index to locate and analyze burned areas. NBR specifically targets post-fire assessments, emphasizing the contrast between NIR and short-wave infrared (SWIR) reflectance, which NDVI cannot capture in severely burned regions [36]. These indices are the most commonly used indices for determining fire severity levels and vegetation regrowth [36,37,38,39,40,41]. These vegetation indices play a critical role in post-fire studies, offering essential tools for tracking vegetation recovery over time [42]. Numerous recent studies use these indices to measure vegetation dynamics across multiple ecosystems, resulting in consistent, geographically wide, and chronologically rich datasets that facilitate landscape-scale assessments of resilience, recovery trajectories, and long-term environmental change [43].

What distinguishes this study is its comparative analysis of post-fire vegetation recovery across four spatially distinct zones within Bosco Difesa Grande, each with a different fire history (unburned, single fire in 2017, single fire in 2021, and repeated fires). By leveraging a multi-year NDVI time series derived from Sentinel-2 imagery and cloud-based analysis on the Google Earth Engine platform, this research provides a high-resolution, long-term perspective on vegetation resilience in a Mediterranean context—an approach that remains underrepresented in the current literature.

In southern Italy, particularly in protected areas such as Bosco Difesa Grande, the frequency and severity of wildfires have increased in recent years, reflecting broader Mediterranean trends. Despite this growing threat, few studies have focused on localized, high-resolution assessments of post-fire vegetation dynamics. This gap limits the effectiveness of ecological recovery planning and adaptive land management. To address this need, our study examines post-wildfire burn severity and vegetation recovery using remote sensing techniques on the Google Earth Engine (GEE) platform, with a comparative analysis of NDVI and NBR indices across multiple burn zones. Bosco Difesa Grande provides an ideal case study due to its frequent fire events, ecological importance, and diverse topography, enabling a comprehensive assessment of fire impacts on vegetation structure. The use of GEE facilitates the efficient processing of large-scale satellite datasets and the generation of consistent, multi-temporal vegetation metrics necessary for tracking post-fire recovery over time [44,45].

Numerous previous studies have quantitatively assessed burn severity and post-fire vegetation regrowth using remote sensing indices. For example, researchers have employed NDVI to monitor early post-fire vegetation trends [30,31], while others have demonstrated the effectiveness of NBR for mapping burn severity in various ecosystems [35,37]. More recent approaches, such as using Random Forest models combined with Tasseled Cap indices, have enhanced the precision of regrowth assessments across different climatic regions [24]. These studies highlight the importance of spectral indices for reliably quantifying the impacts of fire on vegetation structure and recovery trajectories over time.

This study aims to detect post-fire plant changes using NDVI and NBR, as well as analyze regrowth patterns across varied fire severity for Bosco Difesa Grande from 2017 to 2024, capturing both immediate and medium-term changes. Emphasis is placed on analyzing vegetative resilience, an ecosystem’s ability to recover its structure and function after fire disturbance, across gradients of burn severity.

## 2. Materials and Methods

### 2.1. Study Area

**Figure 1:**
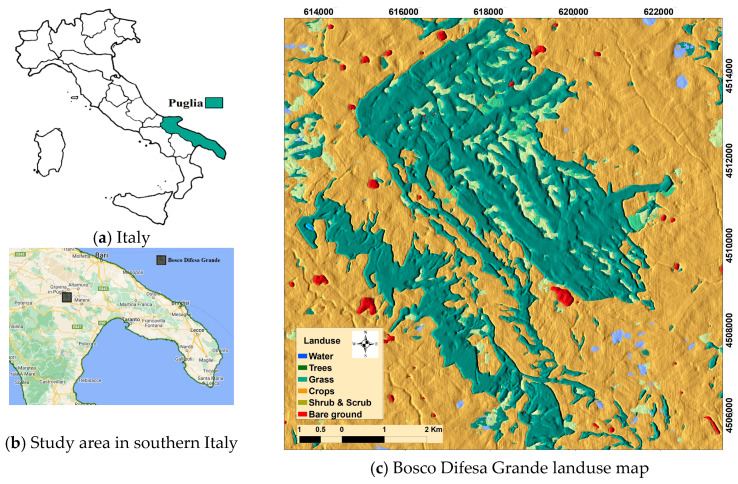
Puglia Region in southern Italy (**a**), Bosco Difesa Grande location in Puglia (**b**), land-use map of the study area (**c**) using Dynamic World V1 (2023).

This study focuses on the Bosco Difesa Grande forest in southern Italy, roughly 6 km south of Gravina. Covering around 1890 hectares (Figure 1), it is one of Apulia’s most important woodland complexes rich in ecosystems, flora, and fauna species of conservation concern [46]. The forest is situated in a hilly landscape within the Bradano River’s catchment area, bordered by the Basentello and Gravina tributaries to the west and east, respectively [47]. The elevation ranges from 245 to 466 m above sea level. This area represents a remnant of the vast mesophytic forest that once covered much of Puglia during the early middle Holocene [48]. Due to its significant ecological value, Bosco Difesa Grande has been designated as a Site of Community Importance, as indicated by the Ministerial Decree of 2000 [47], (Bosco Difesa Grande, Site of Community Importance of Gravina https://puglia.com (accessed on 10 November 2019)). This classification highlights the area’s rich biodiversity, conservation importance, and protected status within the European Union’s Natura 2000 network. The decree imposes regulations to protect the forest from environmental threats, including wildfires, deforestation, and land degradation, while promoting sustainable management and restoration efforts.

**Figure 2:**
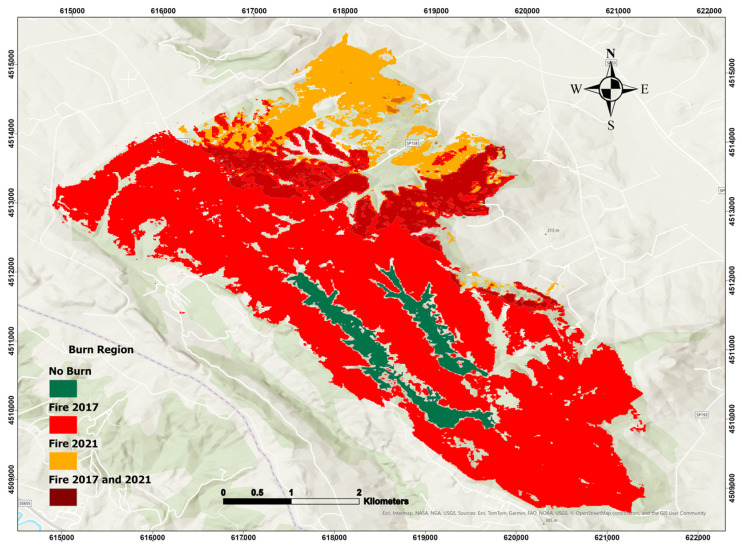
Spatial distribution of classified regions in the study area based on fire history: region with no recorded fire (control), region burned in 2017, region burned in 2021, and region burned in both 2017 and 2021. This classification supports the comparative analysis of vegetation recovery and burn severity.

The study area frequently experiences fires, with several recent small to large burning events impacting the region. Table 1 shows the number and dates of fire incidents in the research area, including the two most recent fire events. The most recent fires, in 2017 and 2021 (Figure 2 and Table 1), have affected many hectares of forest, resulting in significant post-fire consequences for vegetation recovery; therefore, these events became the focus of our analysis.

**Figure 3:**
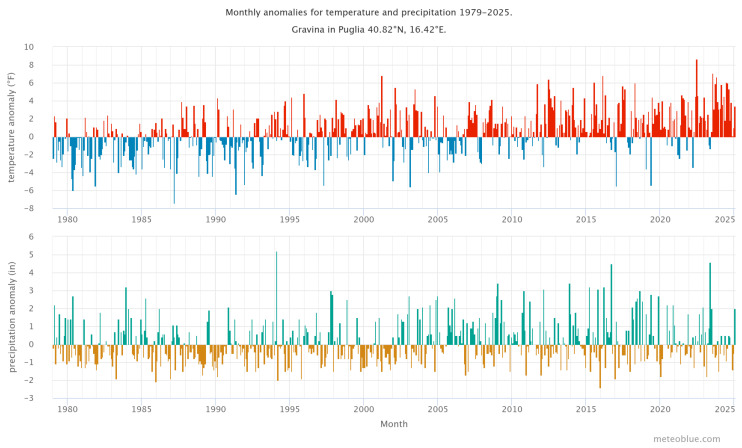
Monthly temperature (F) and precipitation anomalies (in), Climate Change Gravina in Puglia from 1980 to 2025 (source: http://meteoblue.com (accessed on 1 January 2025)).

Climate change has impacted Gravina, Puglia, Italy, creating rising temperatures, altered precipitation patterns, and more extreme weather events reflecting the broader effects of climate change in Southern Italy [49]. In Figure 3, the top graph depicts the temperature anomaly for each month from 1979 to the present, comparing recorded temperatures to the 30-year climatic mean (1980–2010). The red-shaded months have higher temperatures than the blue-shaded months. Warmer months have increased over time, mirroring the overall warming trends related to climate change in Southern Italy. The bottom graph depicts the precipitation anomaly for the same period, displaying monthly rainfall differences from the 30-year average. Green-shaded months reflect wetter-than-average circumstances, whereas brown-shaded months represent drier seasons. These changes emphasize altering precipitation patterns, which, together with rising temperatures, add to the region’s climate unpredictability [49,50]. The climate chart (Figure 3) provides a contextual framework for understanding the climatic parameters that influence burn severity and vegetation regeneration in the Gravina region. The figure depicts rising temperatures and shifting rainfall patterns, which may have an impact on vegetation resilience and regeneration following fire outbreaks. Higher temperatures can exacerbate fire intensity and soil damage, but rainfall variability can have an impact on post-fire soil moisture levels, which are crucial for plant recovery. This climate information helps to set the environmental context for interpreting the vegetation recovery dynamics in the research area [4,51].

**Figure 4:**
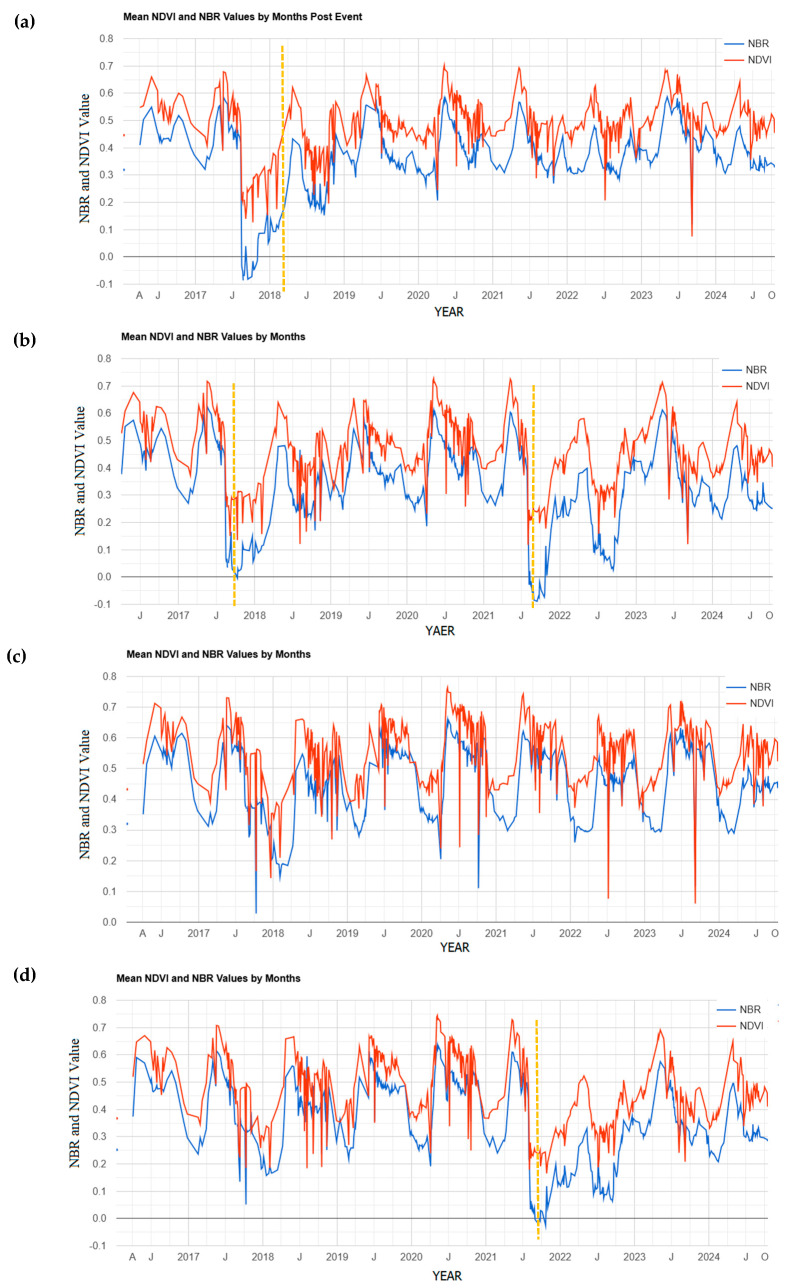
Time series analysis of mean NDVI (Normalized Difference Vegetation Index) and NBR (Normalized Burn Ratio) for four regions classified by fire history: (**a**) region burned in 2017, (**b**) region affected by fires in 2017 and 2021, (**c**) unburned control region, and (**d**) region burned in 2021. The graphs illustrate vegetation response and recovery patterns following fire events. Dashed orange lines indicate the timing of fire events, which correspond to sharp declines in NDVI and NBR. Recovery trends vary by region and fire frequency.

To facilitate a comparative analysis of vegetation recovery trends, the study area was divided into four regions according to their respective fire histories. These include an unburned control area, regions affected by a single fire event (in 2017 or 2021), and a region subjected to two consecutive fire events (in 2017 and 2021). By analyzing NDVI time series across these differently impacted areas, the study assesses how fire frequency and the time elapsed since the last fire influence burn severity and post-fire vegetation regeneration. The spatial distribution of these four regions is illustrated in Figure 4: (a) area burned in 2017, (b) area affected by both 2017 and 2021 fires, (c) unburned control area, and (d) area burned in 2021.

### 2.2. Burn Severity and Vegetation Recovery

To effectively evaluate the extent of fire damage and assess the potential for future destruction, a detailed survey of the region is essential [41,52]. Various mapping techniques have been explored and tested [53], relying on the spectral signature variations of fire-impacted surfaces. Typically, short-wave infrared (SWIR) reflectance increases while red and near-infrared (NIR) reflectance decreases in burned areas [54]. Several reflectance indices derived from digital satellite data are utilized to describe the phytosociological characteristics of vegetation [55]. Recently, cloud-based resources like Google Earth Engine have garnered global attention due to their ability to provide rapid and accurate analysis on several remotely sensed images referred to as time stacks [40,56]. Therefore, GEE was utilized to detect post-fire vegetation burn and recovery in fire-prone environments. For this purpose, we calculated the two most commonly applied indices: normalized difference vegetation index (NDVI and dNDVI) and normalized difference burn ratio (NBR and dNBR) [53,57] (Table 2). The spatial layout and thematic display of the calculated indices were produced with ESRI ArcGIS Pro. To add geographic context, the “World Topographic Map” and “World Hillshade” basemaps—available inside ArcGIS Pro 3.4.0 version—were used as background layers during figure development.

In particular, NDVI can be used to assess vegetation health and dNDVI to measure post-fire vegetation loss and recovery, while NBR and dNBR are more effective for assessing burn severity by capturing fire-induced changes in vegetation and soil properties [58]. Together, these indices provide a comprehensive view of fire impact and post-fire vegetation regeneration. Furthermore, this study looks at the temporal correlations between fire episodes and post-fire vegetation recovery in the Bosco Difesa Grande region, utilizing zonal statistics for each burn severity class in the first, second, and third years after the fire.

One of the most popular change detection approaches is the differentiation of single band indices obtained from multispectral pre- and post-fire images. The change detection analyses examine how to map the impacts of wildland fire on plants using NDVI and NBR. Burn severity can be estimated by subtracting the post-fire value from the pre-fire value [37,59,60]. This yields a measure of absolute change. The difference between the NDVI and the NBR images is calculated so that it can be seen which regions have changed in which direction. In addition, the dNDVI and dNBR indices can also provide information on vegetation regeneration. The dNBR index is very useful to identify burned surfaces and determine burn severity levels [61,62]. One of the most often utilized algorithms for change detection is the distinction of single band indices from multispectral images before and after a fire event. The NBR index considers the NIR and SWIR spectral areas, which are less sensitive to atmospheric influences and may effectively predict the damaged vegetation and the canopy moisture loss (a decrease in NIR regions and an increase in SWIR region in the post-fire scenario) [62]. Additionally, this index might include details on vegetation regeneration. The fire scar will start to reflect a stronger signal in the near-infrared (NIR) section of the spectrum once vegetation regeneration has started since healthy plants reflect significantly in this area of the spectrum because of the characteristics of chlorophyll. As a result, negative dNBR values suggest vegetation regeneration [63,64].

**Table 2:** Index Formula.

Index	Name	Equations	Reference
NBR	Normalized Burn Ratio	(NIR − SWIR)/(NIR + SWIR) NBR = (B08 − B12)/(B08 + B12)	Keeley [65]
NDVI	Normalized Difference Vegetation Index	(NIR − RED)/(NIR + RED) NDVI = (B8 − B4)/(B8 + B4)	Rouse et al. [66]
dNBR	Differenced Normalized Burn Ratio	dNBR = NBR_pre-fire_ − NBR_post-fire_	Key and Benson [67]
dNDVI	Differenced Normalized Difference Vegetation Index	dNDVI = NDVI_pre-fire_ − NDV_post-fire_	Escuin et al. [68]

The NDVI classification pixel range indicates various levels of vegetation density. In general, its values are negative for water bodies, close to zero for rocks, sands, or concrete surfaces, and positive for vegetation, including crops, shrubs, grasses, and forests. In other words, greater NDVI values mean stronger implications for vigorous vegetation greenness [65,66]. NDVI always ranges from −1 to +1, but there is not a distinct boundary for each type of land cover. For example, negative values indicate that value represents water, whereas NDVI values close to +1 represent dense green leaves [30]. However, NDVI values close to zero suggest no green leaves or an urbanized area [29].

NBR is a different band ratio that uses the near-infrared and short-wave infrared bands and is like NDVI. Higher NBR values imply healthy vegetation cover in each area, whereas lower NBR levels indicate bare ground or recently burned areas [31]. NBR is a significant and well-respected method for identifying burned areas in large fire zones [32,33]. The near-infrared (NIR) and short-wave infrared (SWIR) bands are combined in this method.

The spectral bands of the Near Infrared (NIR) and Shortwave Infrared (SWIR) are important for identifying burned areas. NIR emphasizes variations in leaf burn brightness and canopy cover, while SWIR identifies variations in landscape dryness [34]. After a fire, the NIR reflectance drops sharply because of the vegetation being destroyed; nevertheless, the SWIR reflectance rises as a result of the fire removing water-retaining plant cover [35]. The Differenced Normalized Burn Ratio (dNBR) was used to assess burn severity. Thresholds for severity classification were adapted from the European Forest Fire Information Service (EFFIS) [65] (Table 3). According to previous studies, the dNDVI index is divided into six classes to measure burn severity [67] (Table 3). dNBR is more successful at determining burn severity shortly following a fire. dNDVI is beneficial for monitoring post-fire vegetation recovery; however, it may be less effective for discriminating between severe burns. Thus, dNBR was used to indicate burn severity and dNDVI for vegetation recovery. Creating Burn Severity Maps is critical for successful wildfire management and ecological restoration. These maps give essential information about the immediate and long-term effects of fires on different ecosystems. For example, the US National Park Service emphasizes that satellite-derived burn severity maps are vital tools for both fire and resource management, particularly in remote regions [68].

### 2.3. GEE-Based Wildfire Monitoring and Vegetation Recovery Analysis

This study used the Google Earth Engine (GEE) platform to process satellite images. We used the COPERNICUS/S2_HARMONIZED Sentinel-2 Level-2A Surface Reflectance product used in this study was provided by the European Space Agency (ESA), Paris, France, under the Copernicus Programme and accessed via Google Earth Engine, which provides multispectral imagery at spatial resolutions ranging from 10 to 60 m and has a revisit time of about 5 days. Pre-processing included filtering by acquisition date and cloud cover, applying cloud and shadow masking with the QA60 band and the s2cloudless algorithm, and creating composite images for pre- and post-fire periods. Vegetation indices, specifically the Normalized Difference Vegetation Index (NDVI) and the Normalized Burn Ratio (NBR), were calculated using Sentinel-2 bands—B4 and B8 for NDVI and B8 and B12 for NBR—with a spatial resolution of 10 m. B4 in this dataset is in the wavelength of 664.5 nm (S2A)/665 nm (S2B); B8 is NIR in 835.1 nm (S2A)/833 nm (S2B); B12 is SWIR 2 with 202.4 nm (S2A)/2185.7 nm (S2B).

This high spatial resolution allowed for extensive examination of vegetation status and fire severity throughout the research area. All image processing, index calculation, and temporal compositing were carried out within the GEE environment to ensure consistency and scalability. Because the COPERNICUS/S2_HARMONIZED dataset contains pre-scaled surface reflectance values (0 to 1), no additional multiplicative or additive factors were used in vegetation index computations. NDVI and NBR were calculated directly using GEE’s normalized difference approach on reflectance bands.

The post-fire vegetation recovery has been assessed by evaluating NDVI trends using time series analysis [29,69]. Indices were calculated for periods before and after each fire event, and the median values were aggregated into a single raster layer. The period of study is determined by the availability of Sentinel data beginning in 2015.

NDVI trends over time were analyzed using zonal statistics to assess vegetation recovery across different burn severity levels. Burn severity classes were derived from dNBR values and categorized based on widely accepted threshold values (Table 3). The resulting burn severity map included five categories: unburned and very low, low, moderate, high, and very high severity. Using ArcGIS Pro, the classified severity map was used as the burn zones layer, and NDVI raster stacks—covering pre-fire and three years post-fire—served as input value layers. The ‘Zonal Statistics as Table’ tool was employed to extract the median NDVI values for each burn severity class on an annual basis. The resulting statistical tables were exported to Microsoft Excel, where line charts were generated to visualize the temporal trends in NDVI for each severity category.

## 3. Results and Discussion

### 3.1. Time Series of Mean NDVI and NBR

To gain a comprehensive understanding of vegetation changes across the four distinct zones in the study forest, we utilized Google Earth Engine to generate time-series analyses of NDVI and NBR for each section.

A time series analysis of mean NDVI (Normalized Difference Vegetation Index) and NBR (Normalized Burn Ratio) provides valuable insights into vegetation condition and temporal changes in response to fire events [70]. In burned areas, both indices typically show a marked decline immediately following fire, followed by a gradual recovery as vegetation regrows. The sudden drops in NDVI and NBR observed in Figure 4 correspond directly to the timing of fire events, highlighted by dashed orange lines. These declines indicate immediate vegetation damage. The timing and speed of recovery after each drop vary depending on fire frequency, intensity, and local environmental conditions.

Figure 4a,b,d show sharp declines in both NDVI and NBR following fire events, with mean NBR values falling below zero, indicating high burn severity. In Figure 4a, the area experienced a single fire event in August 2017, after which vegetation showed substantial recovery within three years. A slight decline in 2022 does not coincide with any recorded fire and may be attributed to other environmental stressors, such as climate variability.

Figure 4b presents two distinct drops in NDVI and NBR, corresponding to fire events in 2017 and 2021. In both cases, post-fire recovery is observed, with NDVI rebounding more quickly than NBR. Throughout recovery periods, NDVI remains consistently higher than NBR, indicating ongoing vegetation regrowth despite repeated disturbances. The second fire appears more severe, as the NBR value drops further below zero and recovers more slowly, remaining around zero even a year after the event.

Figure 4c represents the unburned control zone, where the time series primarily reflects seasonal and interannual vegetation variability. A reduction in 2018 may be attributed to indirect fire effects: although the area was not directly burned, nearby fires may have influenced vegetation health through smoke, ash deposition, or heat stress—factors known to affect photosynthetic activity and plant moisture [71]. Previous research has shown that high-severity burns can increase daily maximum temperatures and vapor pressure deficits in surrounding unburned areas, altering local microclimates [65]. Figure 4d shows the region burned in 2021. Following the fire, NDVI gradually increases, indicating a slow but steady recovery process. This upward trend reflects post-fire succession, seed germination, and resprouting of surviving vegetation. However, the slower recovery rate may be due to factors such as burn severity, soil degradation, or unfavorable environmental conditions limiting regeneration [66].

### 3.2. Burn Severity

**Figure 5:**
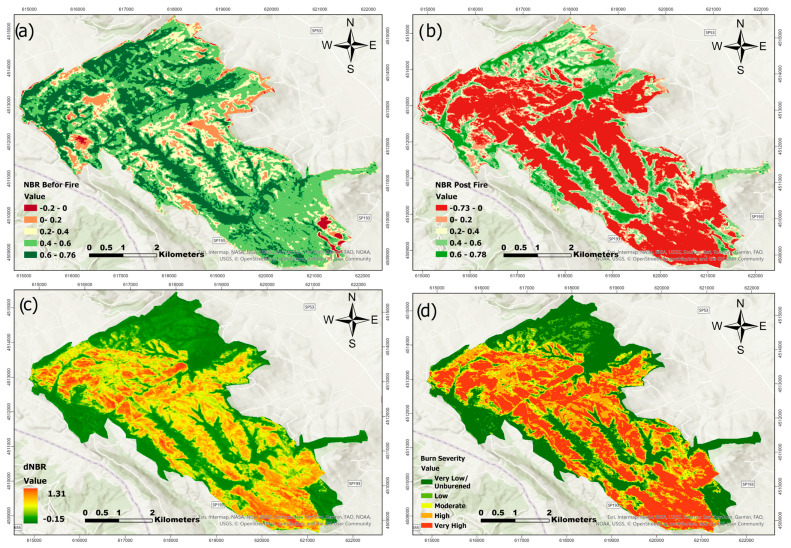
NBR before fire (**a**), NBR post-fire (**b**), dNBR (**c**), and Burn severity classification (**d**) for fire event in August 2017.

**Figure 6:**
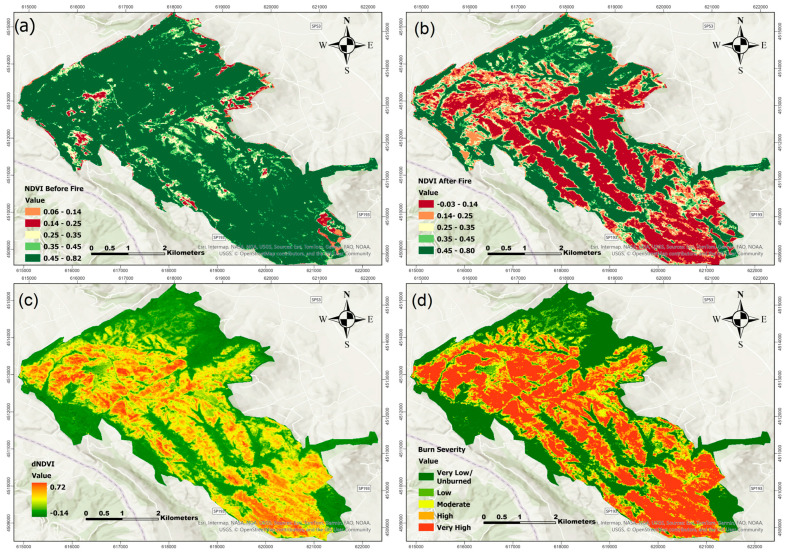
NDVI before fire (**a**), NDVI post-fire (**b**), dNDVI (**c**), and Burn severity classification (**d**) for fire event in August 2017.

Burn severity for the two fire events was assessed using the differenced Normalized Burn Ratio (dNBR) and differenced Normalized Difference Vegetation Index (dNDVI). For each event, pre- and post-fire satellite imagery was processed to calculate dNBR and dNDVI, and the results were classified into severity categories based on the thresholds outlined in Table 3. Figure 5 and Figure 6 show the burn severity results from the August 2017 fire event. Figure 5 depicts the NBR-based analysis. Panel (a) displays the pre-fire NBR image, suggesting good plant conditions, while panel (b) depicts the post-fire NBR image, which shows a decline in vegetation reflectance due to fire. Panel (c) shows the dNBR, which highlights places with considerable spectral shift, while panel (d) shows the classified burn severity map, which distinguishes between zones of low, moderate, and high severity. Figure 6 has the same structure as the NDVI-based analysis of the 2017 fire: panels (a) and (b) show NDVI values before and after the fire, respectively; panel (c) depicts the calculated dNDVI; and panel (d) provides the appropriate burn severity classification.

**Figure 7:**
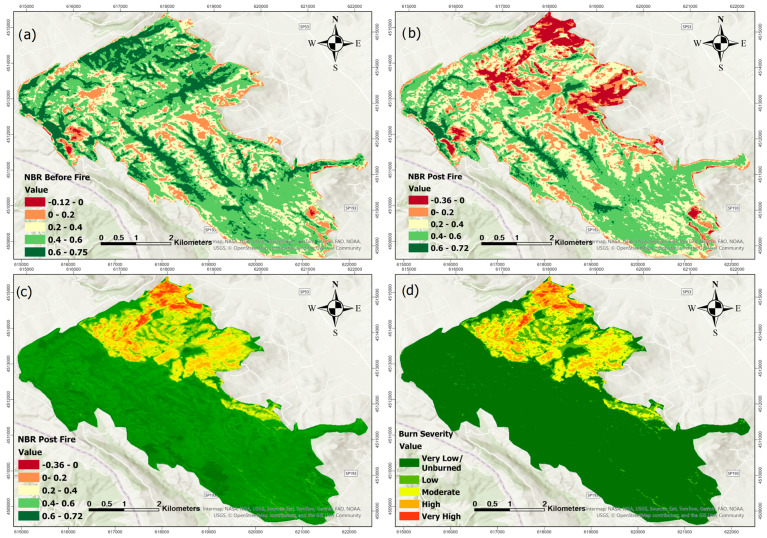
NBR before fire (**a**), NBR post-fire (**b**), dNBR (**c**), and Burn severity classification (**d**) for fire event in July 2021.

**Figure 8:**
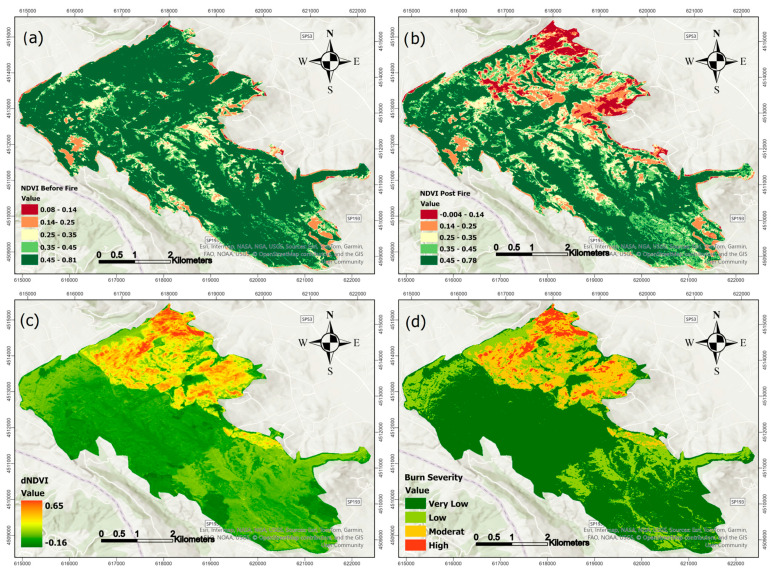
NDVI before fire (**a**), NDVI post-fire (**b**), dNDVI (**c**), and Burn severity classification (**d**) for fire event in July 2021.

Figure 7 and Figure 8 illustrate the same process for the July 2021 fire. In Figure 7, NBR-based panels show similar trends, with strong reductions in post-fire values and a clearly defined dNBR map (panel c) leading to a severity classification (panel d). Figure 8 presents the NDVI-based analysis for the 2021 event, highlighting vegetation loss and recovery patterns consistent with those observed through NBR, though with some spatial variation in boundary areas.

As observed in Figure 5, Figure 6, Figure 7 and Figure 8, there are noticeable differences between the dNBR and dNDVI outputs due to their distinct spectral sensitivity and response to fire impacts. dNBR provides more detailed differentiation in areas with higher burn intensity, while dNDVI is often more responsive to moderate or low-severity burns and partial canopy changes. These complementary indices together offer a more robust assessment of fire effects across varying vegetation types and burn intensities [68].

### 3.3. Burn Severity and Vegetation Regrowth

**Figure 9:**
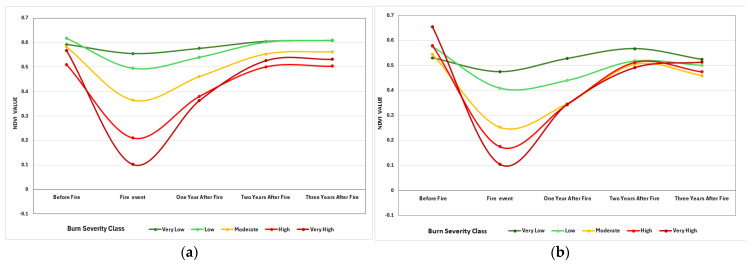
Vegetation recovery in each burn zone after one year, two years, and three years of fire in the area burned in August 2017 (**a**) and the area burned in July 2021 (**b**) using zonal statistics.

One year after the 2017 fire (Figure 9a), NDVI begins to increase at all burn severity levels; however, the rate of recovery varies. Areas with extremely low and low severity recover faster, with NDVI values approaching pre-fire levels. Moderate severity zones exhibit a gradual increase while remaining below their original values. High and very high severity zones have a delayed recovery, with NDVI remaining much lower. Vegetation recovery is progressing at all severity levels, with low and very low severity areas approaching pre-fire NDVI levels, moderate severity improving but lagging, and high to very high severity recovering the slowest.

We expect a similar trend for the 2021 fire event. Recovery rate diminishes with burn severity; very low and low-severity areas recover quickly, approaching pre-fire NDVI levels, but moderate zones show consistent improvement. High and extremely high severity areas show protracted recovery, with NDVI still lagging three years later. A slight decrease in NDVI across all burn severity classifications after three years could be attributed to a variety of variables and require further research. The decline in NDVI across all burn severity classes after three years could be attributed to climatic variability (e.g., drought or seasonal shifts), post-fire soil degradation that reduces nutrient availability, natural vegetation succession where early colonizers decline, or secondary disturbances such as pests, disease, or human activities (e.g., grazing or logging). These factors cumulatively impede long-term vegetation regrowth, resulting in the observed NDVI reduction [67,68].

### 3.4. Vegetation Recovery Evaluation in Burned and Nonburned Regions

**Figure 10:**
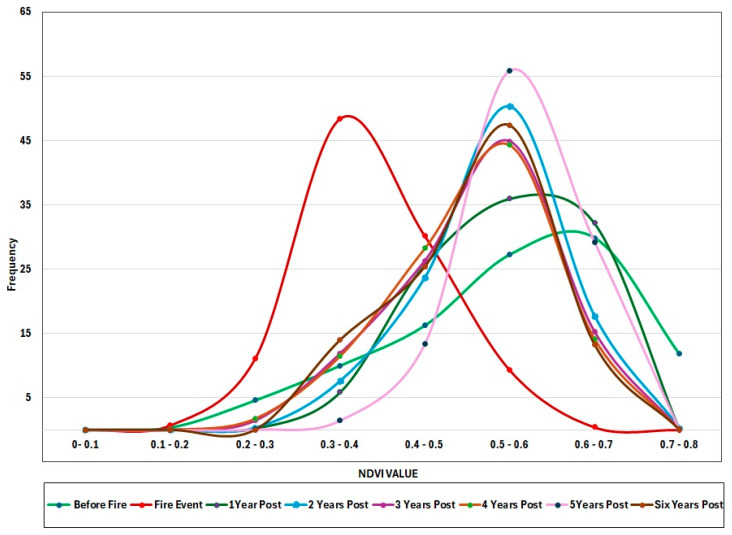
Yearly Frequency Distribution of NDVI Values (2016–2024) in the region hit by fire in 2017.

Before the fire in August 2017, NDVI values were high, suggesting dense and healthy vegetation, with the peak in the higher NDVI range. In the event of a fire, the peak changes to the lower NDVI range, representing fire-damaged vegetation. One year after the fire, NDVI remained low, with modest recovery and varying regrowth over the area. NDVI gradually increased over 2–6 years; however, values remained below 0.7, indicating that recovery was incomplete. Instead, NDVI stabilized between 0.4 and 0.6, indicating a shift from dense forest to mixed vegetation. This shift suggests that following major fires, forests may not fully rebound, potentially resulting in long-term vegetation change (Figure 10). Multiple factors could potentially explain the observed changes in NDVI after large fires, warranting further investigation. Fire severity and frequency are important, as recurrent or high-intensity burns can change species composition, favoring fire-resistant shrubs and grasses over thick forests [69]. Soil deterioration and nutrient availability changes following a fire can further hinder forest recovery [70]. Furthermore, climate change-induced alterations in temperature and precipitation patterns may make circumstances less conducive for forest regrowth, resulting in chronic mixed vegetation states [51]. Hydrological changes, such as decreased soil moisture retention, can also lead to delayed or incomplete forest regeneration [71,72]. Finally, human activities such as land use change, logging, and grazing can stymie natural regeneration efforts. These combined considerations indicate that, in certain circumstances, forests may not fully recover from severe wildfires, potentially leading to long-term ecological shifts.

**Figure 11:**
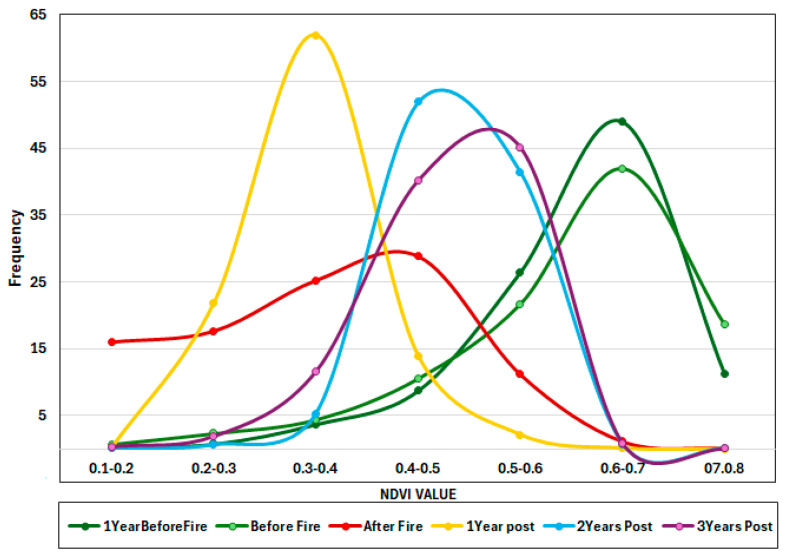
Yearly Frequency Distribution of NDVI Values (2021–2024) in the region hit by fire in 2021.

Before the fire in July 2021, NDVI measures were high, with a peak over 0.7, indicating dense, robust vegetation, a mature forest. The NDVI values drop to the lower range (0.2–0.4), indicating serious vegetation loss and degradation. One year after the fire, the NDVI stays low but gradually increases. The peak is broader, implying that recovery rates vary across locations. Two years after the fire, the NDVI climbs further, reaching a peak of 0.5–0.6, suggesting moderate regeneration. Three years post-fire, the NDVI peak remains between 0.4 and 0.5, indicating that vegetation is stabilizing in the lower NDVI range. The absence of NDVI readings above 0.7 indicates that the forest has not completely recovered (Figure 11).

**Figure 12:**
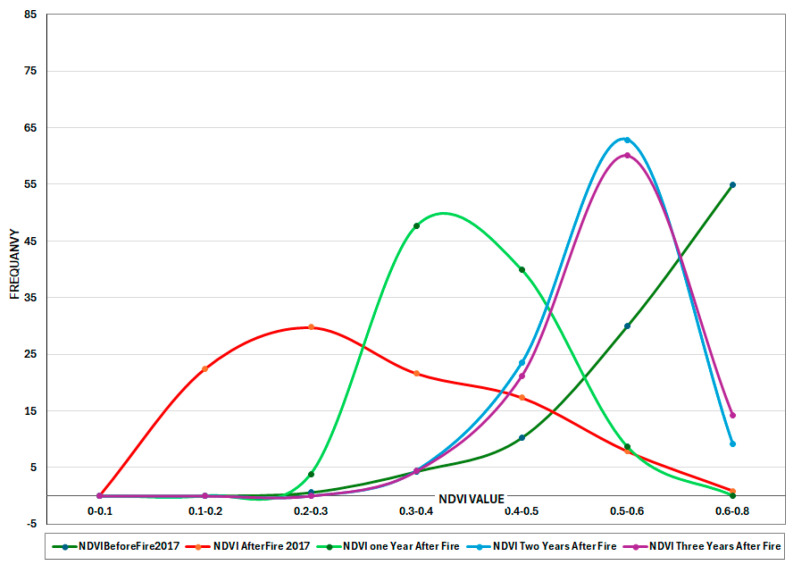
NDVI time series for overlapped burned area in August 2017.

Overlapped burned areas from August 2017 and 2021 are shown in two separate charts for each fire occurrence (Figure 12). In this area before the fire in 2017, NDVI values were high, peaking at 0.6–0.7, suggesting dense growth and a healthy forest. After the fire, NDVI readings changed to lower values (below 0.4), indicating significant plant loss and increasing bare ground. One year after the fire, NDVI levels begin to recover, increasing toward 0.5.

**Figure 13:**
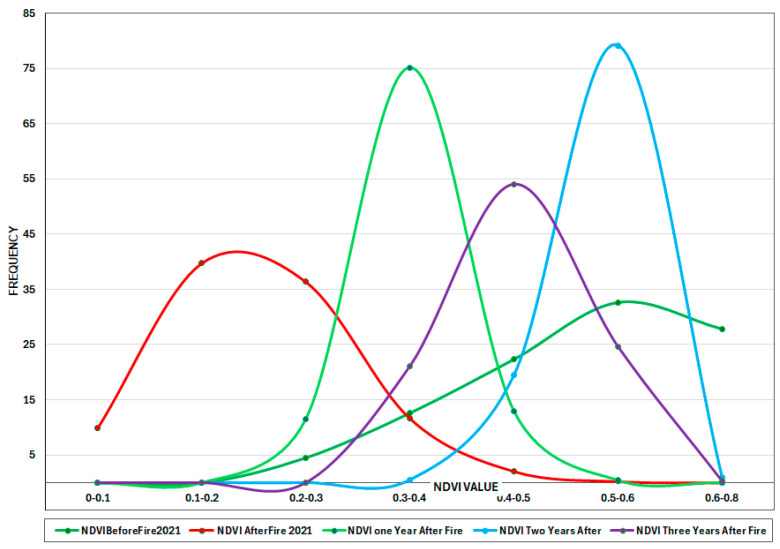
NDVI time series for overlapped burned area in 2021.

Two years post-fire, NDVI continues to rise, with a peak at 0.55–0.6, indicating considerable vegetation recovery. NDVI values three years after fire peak around 0.6, bringing the values closer to pre-fire conditions but still lacking readings above 0.7. The greatest NDVI values (above 0.7) remain absent after the fire, indicating that the canopy has not fully recovered. The shift in peak NDVI values suggests a change in vegetation type, such as shrubland or open woodland, rather than dense forest. If this pattern continues, the post-fire ecology may stabilize with lower vegetation density than before the fire (Figure 12). After the July 2021 fire in this same area (Figure 13), similar trends were observed across both fire events, reinforcing the idea that high-severity fires can hinder full forest recovery. This pattern suggests that repeated intense fires may alter ecosystem dynamics, potentially leading to long-term shifts in vegetation structure and composition.

**Figure 14:**
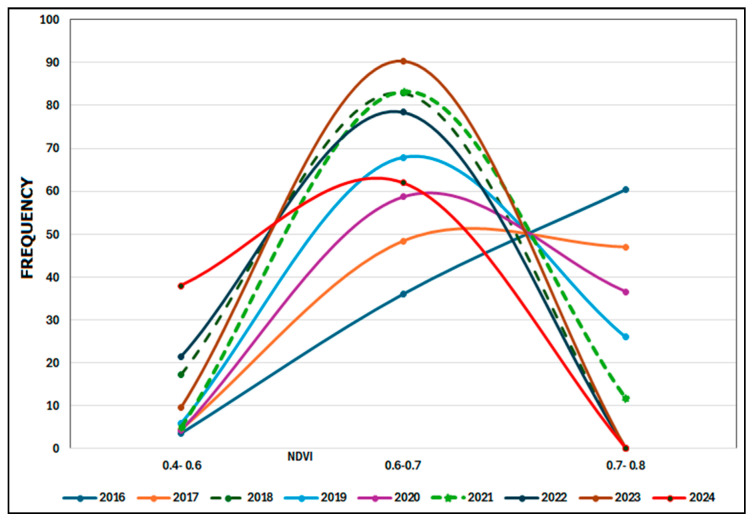
Yearly frequency distribution of NDVI values for the non-burned region in the period of (2016–2024).

**Figure 15:**
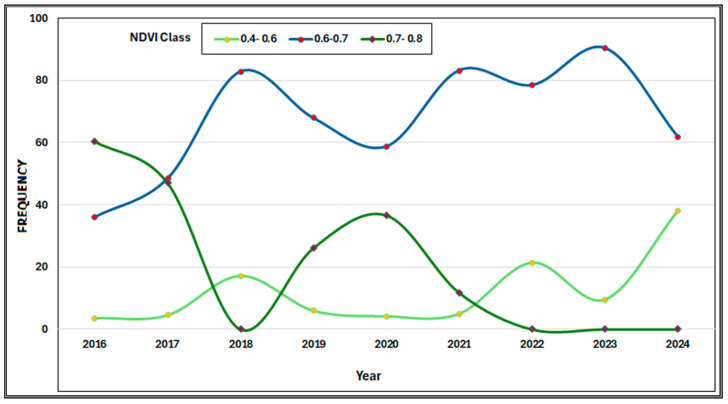
Temporal trends in NDVI frequencies in three value classes for the non-burned region in the period of (2016–2024).

**Figure 16:**
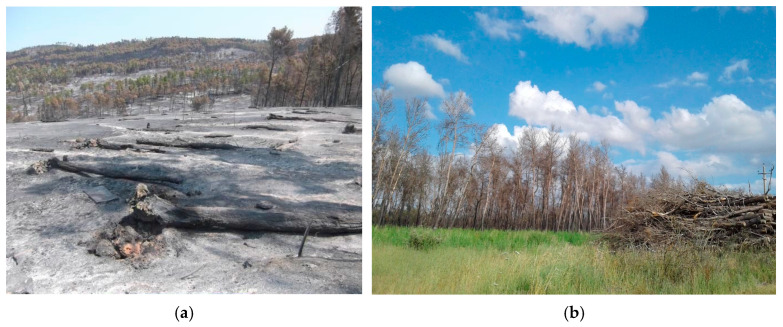
After the fire event in 2017 (**a**), and two years after the fire in the burned area (**b**).

The NDVI values are divided into three groups: 0.4–0.6, 0.6–0.7, and 0.7–0.8. Most years have a bell-shaped distribution, peaking in the 0.6–0.7 NDVI range, indicating that most of the vegetation falls into this category (Figure 14). The highest value of NDVI (0.7–0.8), which represents the densest and healthiest vegetation, occurs in 2016, 2017, and 2020, and after 2020 it declines to almost zero for the rest of the period (Figure 15). The 0.4–0.6 NDVI class rises in some years (2018, 2022, and 2024), which may indicate periods of vegetative stress, degradation, or environmental disturbances. The chart illustrates that high NDVI levels (0.7–0.8) are decreasing while moderate NDVI values (0.6–0.7) are increasing. A decline in high NDVI values (0.7–0.8) combined with an increase in moderate NDVI values (0.6–0.7) may suggest a transition from a tree-dominated ecosystem to one that includes more shrubs or mixed vegetation. This pattern indicates a decrease in thick, healthy vegetation and an increase in thinner or stressed vegetation [20,71]. Figure 16a displays a photo taken soon after the 2017 fire, whereas Figure 16b shows a photograph taken two years later, demonstrating the area’s post-fire recovery.

Overall, the comparative analysis across the four fire history zones reveals key differences in vegetation recovery trajectories. Unburned control zones remained stable with high NDVI values, while single-burn areas (2017 or 2021) exhibited moderate recovery trends. Notably, the zone affected by both the 2017 and 2021 fires showed the slowest and least complete recovery, with NDVI values failing to return to pre-fire levels even three years post-fire. These patterns highlight the compounding effects of repeated fire events and underscore the role of burn severity in shaping post-fire ecological responses.

## 4. Conclusions

Forest fires are a major cause of NDVI decline in the Apulian and Basilicata regions, resulting in severe vegetation loss. Our research in Bosco Difesa Grande revealed the efficacy of remote sensing techniques, specifically the dNBR and dNDVI indices, in measuring wildfire impacts and post-fire vegetation recovery. An analysis of NDVI time series found that NDVI values fell rapidly shortly after wildfire episodes, indicating extensive plant destruction. One year after the fire, initial signs of regrowth were seen, with modest rises in NDVI values; nevertheless, full recovery to pre-fire levels (0.7–0.8) had not yet been achieved.

The constant trend across the NDVI time series, which includes vegetation loss, partial recovery, and signs of ecosystem shifts toward less dense vegetation types such as shrublands, demonstrates the long-term effects of wildfire disturbances on the studied area.

These findings highlight the importance of accurate and continual post-fire vegetation monitoring in guiding land management decisions. Targeted reforestation efforts, adaptive land management, and continuous ecosystem restoration measures will be critical for increasing resilience and maintaining ecological stability in fire-prone Mediterranean regions.

Our spatially explicit comparison across fire histories revealed that areas burned twice exhibited the lowest vegetation recovery, suggesting cumulative fire damage can hinder forest regeneration. In contrast, single-burn areas showed partial but progressive regrowth, while unburned zones remained ecologically stable. These insights are crucial for prioritizing restoration efforts in Mediterranean regions where repeated wildfires may shift ecosystems toward lower vegetation density states.

## Figures and Tables

**Table 1:** Historical fire events in the study area.

Fire Location	Date: Month/Day/Year	Area (ha)
Bosco Difesa Grande-Rifessa Pantone	07/12/2010	0.39
Bosco Difesa Grande-Rifessa Pantone	07/12/2010	0.918
Bosco Comunale	06/25/2011	1.63
Bosco Comunale Difesa Grande	06/29/2011	19.93
Bosco Comunale Difesa Grande	07/10/2011	27.80
Bosco Comunale Difesa Grande	06/30/2012	12.95
Bosco Comunale Difesa Grande	06/30/2012	16.34
Bosco Difesa Grande	08/15/2013	7.14
**Difesa Grande**	**08/12/2017**	**24.13**
**Difesa Grande**	**08/12/2017**	**44.30**
**Difesa Grande**	**08/12/2017**	**1240.25**
**Difesa Grande**	**07/28/2021**	**935.67**

**Table 3:** Approximate dNBR and dNDVI burn severity levels.

dNBR < 0.100	dNDVI < 0.07	Very Low/Unburned
0.100–0.255	0.08–0.20	Low
0.256–0.419	0.21–0.33	Moderate
0.420–0.660	0.34–0.44	High
dNBR > 0.660	dNDVI > 0.44	Very High

## Data Availability

The original contributions presented in this study are included in the article. Further inquiries can be directed to the corresponding authors.
